# Strategies to preserve the reproductive future of women after cancer

**DOI:** 10.5935/1518-0557.20140087

**Published:** 2014

**Authors:** Bruno R. de Carvalho, Jhenifer K. Rodrigues, Jacira R. Campos, Adelino A. Silva, Ricardo M. Marinho, Ana Carolina J. S. Rosa e Silva

**Affiliations:** 1 GENESIS, Center for Assistance in Human Reproduction, Brasília, DF, Brazil; 2 Pró-Criar Reproductive Medicine, Belo Horizonte, MG, Brazil; 3 Service of Human Reproduction, Department of Gynecology and Obstetrics, Faculty of Medicine of Ribeirão Preto, University of São Paulo, Ribeirão Preto, SP, Brazil; 4 Brazilian Oncofertility Consortium

**Keywords:** Fertility preservation, oncofertility, ovarian tissue cryopreservation, oocyte cryopreservation, embryo cryopreservation, in vitro fertilization

## Abstract

Malignant and cardiovascular diseases are the main causes of death in Brazil. Estimates for 2013 predict the occurrence of 189,150 new cases of cancer in Brazilian women. With advanced detection tools, patients are diagnosed and treated for cancer at a younger age and are more likely to survive. The cytotoxic action of chemotherapeutic agents and radiotherapy very frequently implies serious damage to the gonads, and consequences due to the hypoestrogenism, such as osteoporosis, infertility and premature ovarian failure, are expected. Oncofertility, then, appears as a new area of reproductive medicine, which is dedicated to the development of strategies for the reduction of therapeutic sequels in cancer survivals, ultimately aiming the maintenance of their quality of life and the possibility of biological maternity. This article aims to present an overview of possible options for female fertility preservation after cancer and future perspectives in oncofertility.

## INTRODUCTION

Malignant and cardiovascular diseases are the main causes of death in Brazil. Estimates for 2013 predict the occurrence of 189,150 new cases of cancer in Brazilian women (excluding non-melanoma skin cancers), being breast, cervical and colorectal cancers expected to harm approximately 87 thousand of those patients. Brazilian cancer databases estimate that approximately 3% of all malignancies will occur among patients aged less than 19 years, what leads to more than 5,600 new cases of cancer in this population, detaching leukemias, lymphomas and central nervous system tumors (INCA, 2011).

With advanced detection tools, patients are diagnosed and treated for cancer at a younger age and are more likely to survive. Fortunately, while the incidence of cancer in the world increases by 0.3% each year, cure rates increases around 0.6% annualy. The overall decline in mortality rates due to cancer during childhood was approximately 40% between 1975 and 1995 (Ries *et al.*, 1999). Thus, nowadays the overall five-years survival rates vary from 60% to 91% for female breast cancer and can reach 98% for Hodgkin’s lymphoma (Gosden, 2009; INCA, 2011).

According to the American Cancer Society, it is estimated that approximately 5% of female cancer survivals now living in the United States are younger than 40 years of age and that, in nine years, this population will be of approximately 9.2 million people, which will mean a shift of 30% (American Cancer Society, 2012). Considering this scenario where hundreds of thousands of girls and women with cancer are submitted to chemotherapy and radiotherapy, previous research from Blatt in 1999, estimated that in 2010 one in every 250 adults was going to be a cancer survivor during childhood (Blatt, 1999).

The cytotoxic action of chemotherapeutic agents and radiotherapy very frequently implies serious damage to the gonads (The Practice Committee of the American Society for Reproductive Medicine, 2013). Despite the fact that temporary or permanent ovarian failure will depend on several factors, such as drugs and doses administered, route of administration and age at the time of treatment, long term consequences due to the hypoestrogenism, such as osteoporosis, infertility and climacteric symptoms, may be expected.

This context requires efforts for the reduction of therapeutic sequels in cancer survivals, ultimately aiming the maintenance of their quality of life, which instinctively includes the possibility of biological maternity. Oncofertility appears in this scenario as a new area of reproductive medicine, which is dedicated to the development of new strategies founded to ensure the reproductive future of cancer patients. Several options, such as pharmacological protection of the ovaries and cryopreservation of embryo, oocyte and ovarian tissue (Rosa e Silva *et al.*, 2008; Jeruss & Woodruff, 2009; Cardoso *et al.*, 2012; Reddy & Oktay, 2012) are available to preserve fertility in female cancer patients. This article aims to present an overview of those options and the perspectives in oncofertility.

## GONADAL DAMAGE

The cytotoxic action of certain drugs on the gonads is a real fact, but it is still not known on which cell type it occurs, contributing to greater aggressiveness in the depletion of primordial follicles or in the prevention of follicular maturation. The structural and functional proximity between granulosa cells and oocyte makes it difficult to establish the exact target of those drugs, but it seems obvious that damage to any follicular structures would lead to impairment and dysfunction of the whole system of follicle development (Gradishar & Schilsky, 1989).

Alkylating agents such as cyclophosphamide, mustard L-phenylalanine, chlorambucil, nitrosoureas, melphalan, busulfan and procarbazine may cause irreversible damage to the gonads as a consequence of chromosome deletions and prevention of the synthesis of essential cellular proteins secondary to chemical interactions with DNA. The injuries may range from a quantitative reduction of follicular apparatus to gonadal fibrosis. Other agents such as 5-fluorouracil, methotrexate, vincristin, bleomicyn, dactinomycin, etoposide and doxorubicin may also cause damage, but it is not always irreversible (Table 1) (Shamberger *et al.*, 1981; Blumenfeld *et al.*, 1999; Lee *et al.*, 2006).

**Table 1:** Risk of female gonadotoxicity of various antineoplastic agents (modified from Pentheroudakis *et al*., 2010; Christinat & Pagani, 2012)

	High risk (permanent amenorrhoea in > 80% of exposed women)	Intermediate risk (permanent amenorrhoea between 20% and 80% of exposed women)	Low risk (permanent amenorrhoea in < 20% of exposed women)	Risk not established
Single agents	Cyclophosphamide Busulfan Melphalan Chlorambucil Dacarbazine Procarbazine Ifosfamide Thiotepa	Anthracyclines Cisplatin Carboplatin Ara-C (cytarabine)	Methotrexate Bleomycin 5-Fluorouracil Actinomycin-D Vinca alkaloids Mercaptopurine Etoposide Fludarabine	Taxanes Oxaliplatin Irinotecan Monoclonal antibodies Tyrosine-kinase inhibitors
Combined agents and radiotherapy	Nitrogen mustard High-dose cyclophosphamide/busulfan and haemopoietic stem cell transplantation Ovarian irradiation CMF, CAF, CEF ×6 in women < 40 years	CMF, CAF, CEF × 6 in women 30-39 years AC, EC × 4 in women > 40 years	ABVD CMF, CEF, CAF × 6 in women < 30 years MF CHOP, CVP Protocols for acute myeloid leukemia, acute lymphoid leukemia AC ×4 in women < 40 years	

AC = doxorubicin + cyclophosphamide; CAF = cyclophosphamide + doxorubicin + fluorouracil;

CEF = cyclophosphamide + epirubicin + fluorouracil; CMF = cyclophosphamide + methotrexate + fluorouracil;

MF = methotrexate + fluorouracil; EC = epirubicin + cyclophosphamide;

CHOP = cyclophosphamide + doxorubicin + vincristine + prednisolone;

CVP = cyclophosphamide + vincristine + prednisone; ABVD = adriamycin + bleomycin + vinblastine + dacarbazine.

Studies show that the occurrence of premature ovarian failure (POF) after alkylating chemotherapy are observed in rates of 100%, 50% and 13% among patients older than 30 years, aged 20 to 30 years, and younger than 20 years, respectively, demonstrating that the younger the patient is treated, the lower the damage caused to her ovaries (Blumenfeld *et al.*, 2000). However, other studies have observed that young girls submitted to chemotherapy during childhood and presenting normal pubertal development are likely to present higher FSH levels in comparison to healthy girls, confirming some damage (Rosa e Silva *et al.*, 2007).

Radiotherapy leads to POF in more than 60% of women submitted to doses higher than 300 cGy (Schüffner *et al.*, 2003). With a pelvic exposure to 4 Gy, irradiation is lethal for the oocytes, and the exposure to 5 to 10.5 Gy leads to ovarian failure in 97% of patients (Sanders *et al.*, 1996; Howell & Shalet, 1998).

In addition to ovarian aggression, there may also be injury of uterine vascularization and development (Holm *et al.*, 1999).

## FERTILITY PRESERVATION

The early diagnosis and adequate treatment have been increasing the survival rates of cancer patients. Although surviving adults tend to be more interested in fertility preservation, it is important to remember that the bulk of options should be made available to young cancer patients such as prepubertal girls and also patients who require immediate treatment.

Health professionals must be prepared to discuss fertility with their patients and family regardless the patients’ age or marital status, and always before oncologic therapy is started, so that they may understand the possible consequences of cancer treatment and then decide to preserve or not their fertility, and which strategy better attend to their fertility preservation needs.

### 1. Pharmacological protection of the ovaries

Pharmacological protection of the ovaries by the use of GnRH analogues is controversial. The strategy intends to simulate prepubertal state (i.e., ovaries in a resting state) (Kwon & Case, 2004), once previous data demonstrated lower gonadal damage in girls younger than 15 years treated for Hodgkin’s disease (Blumenfeld *et al.*, 1996). The mechanisms by which protection may occur have not been well established since primordial follicles are not expected to depend on the action of gonadotropins.

Also, even though some studies have suggested the participation of GnRH in follicular physiology from the primary stage to atresia (Clayton *et al*., 1992; Whitelaw *et al.*, 1995) and the expression of their receptors in granulosa-luteal cells is evident (Peng *et al.*, 1994), there are no sufficient data proving its action directly on primordial follicles. One theory proposes a direct action of the analogues on the ovaries in an earlier phase of folliculogenesis by inhibition of mitotic activity (Ataya *et al.*, 1988; Ataya & Moghissi, 1989; Leung *et al.*, 2003), and, then, conferring ovarian protection since the cytotoxicity of the chemotherapic drugs are more intense on cells in a process of overt division.

Other possibilities for the role of GnRH analogues in ovarian protection, according to Blumenfeld, are ovarian perfusion reduction due to hypoestrogenic status, an anti-apoptotic effect mediated by esfingosine-1 phosphate and the preservation of pluripotent germ cells supposedly existent in the ovary (Blumenfeld, 2004).

Blumenfeld *et al.* detected a reduction from 61% to 6.7% in the incidence of POF in women submitted to chemotherapy combined with GnRH analogues (Blumenfeld *et al.*, 1996), and recently confirmed the efficacy of such a strategy with a trial in which 96.9% of the patients resumed regular menses, opposed to only 63% of women submitted to chemotherapy without protection (Blumenfeld *et al.*, 2008). Recchia *et al.* had also obtained evidence of gonad protection after resumption of menstrual regularity in 86% of patients who had taken gosserrelin in combination with chemotherapy for breast cancer (Recchia *et al.*, 2002).

Although some authors suggest this beneficial effect, the only published trial, which included exclusively patients with advanced stages of Hodgkin lymphoma specifically treated with BEACOPP (bleomycin, etoposide, adriamycin, cyclophosphamide, vincristine, procarbazine and prednisone), demonstrated that there was no gonadal protective effect in the use of GnRH analogues during chemotherapy (Behringer *et al.*, 2010). However, the overall literature related to this issue suggest that the administration of GnRHa before and during chemotherapy is associated with an absolute reduction in the incidence of early menopause of nearly 20% (Del Mastro *et al.*, 2011) and higher rates of ovulatory cycles reestablishment, but no impact in reproductive potential have ever been proved (Bedaiwy *et al.*, 2011).

Even though there are no definitive data conferring ovarian chemoprotective effect to GnRH analogues, that drugs present a second beneficial effect on women submitted to chemotherapy, since they may present altered menstrual flows, especially hypermenorrhea and menorrhagia secondary to the common thrombocytopenia induced by chemotherapeutic agents. Suppression of the hypothalamus-pituitary-ovary axis causing temporary chemical castration may prevent such disorders. Care must be taken regarding bone mass, which is supposed to diminish after prolonged hypoestrogenic states; add back therapy will probably be advisable after six months of use or for extremely symptomatic individuals (Rosa e Silva *et al.*, 2008).

Finally, studies on other drugs, like sphingosine-1-phosphate - an apoptosis-inhibitor - or its mimetic FTY720, have suggested the possibility of gonadal protection by local injection near chemotherapy in mice (Hancke *et al.*, 2007) and radiation exposition in primate and human ovaries (Zelinski *et al.*, 2011), supporting a promising treatment option in the future.

### 2. Ovarian transposition

Ovarian transposition consists of surgical pexis of the gonads outside the pelvis, aiming to remove them from localized irradiation field . In a previous report, a small series in which laparoscopic oophoropexy was performed before pelvic irradiation for Hodgkin’s disease, it was effective in preserving gonadal function, even if associated with low doses of chemotherapy (Williams *et al.*, 1999). According to Clough *et al.*, the rates of short-term success may reach 100% in women aged less than 40 years and when low doses of irradiation are applied (Clough *et al.*, 1996), nevertheless POF may reach more than 80% after 35 months of the procedure (Kwon & Case, 2004). Also, it is only indicated if the patient is going to be submitted exclusively to radiotherapy with no chemotherapy associated.

For the reasons above, ovarian transposition is nowadays misused and reserved for isolated radiotherapy candidates, since maintaining vascular pedicle does not prevent the adverse action of the antineoplastic agent in average or high doses. As an important limitation of the method, the possibility of adverse vascular twisting or stretching during transposition may cause flow occlusion and also complicate with gonadal failure (Rosa e Silva, 2006).

### 3. Embryo cryopreservation

Embryo cryopreservation is the most commonly adopted method of cryotechnology in oncofertility. Rates of embryo survival after thawing reach up to 80% (Michelmann & Nayudu, 2006), followed by implantation and clinical pregnancy rates similar to those obtained for fresh embryos. According to the last statistics published by REDLARA (Latin America Assisted Reproducion Network) in 2010, pregnancy rates from fresh and frozen embryos transferred were approximately 36.5 and 22.0%, respectively. However this data are opposed to those reported in a meta-analysis published by Roque *et al.* were elective frozen-thawed embryo cycles resulted slightly better on going and clinical pregnancy rates in comparison to fresh embryos cycles. These authors believe that probably reports of lower pregnancy rates in frozen embryo cycles may be due to embryo quality, once the better embryos are always transferred in the fresh cycle, and not always the remaining embryos are top quality before freezing (Roque *et al.*, 2013).

In any way the limitations of embryo cryopreservation as a strategy for fertility preservation are the need to delay antineoplastic treatment in 2 to 6 weeks, since it requires follicular stimulation (Lee *et al.*, 2006), the high costs, especially those related to gonadotropic ovarian stimulation, the risk of ovarian hyperstimulation syndrome, a potentially serious event and relative contra-indication in patients with estrogen depend tumors (Kwon & Case, 2004; Oehninger, 2005). In relation to the delay in oncologic treatment due to the period of ovarian hyperstimulation, many protocols have been suggested starting induction in different cycle phases, what minimize treatment postponement (von Wolff *et al.*, 2009; Sönmezer *et al.*, 2011).

For hormone-dependent cancers, aromatase inhibitor letrozole has been documented as an alternative to traditional hormonal stimulation, without evolution of malignancies on a short-term basis. Perspectives are centered on the reduction of ovarian estrogen production when the use of letrozole is started before and maintained during stimulation with gonadotropins (Oktay K, 2005; Azim & Oktay, 2007). Similarly, good results have been demonstrated with tamoxifen stimulation, generating positive expectations for breast cancer patients (Oktay *et al.*, 2003). In the case of endometrial neoplasias, protection can be obtained by the use of the intrauterine system with levonorgestrel (Juretzka *et al.*, 2005).

Beside all this, there is also a matter of a second person involved, as it requires seminal samples for fertilization, what in many cases may be a deterrent, because the patient does not have a partner or because the patients autonomy for future conception will always be limited by the partner.

### 4. Cryopreservation of Mature Oocytes

Over the last few years there has been a marked refinement on the cryopreservation of mature oocytes. Despite osmotic changes, damage to the zona pellucida, membrane, cytoskeleton, microtubules and organelles, rupture of cortical granulations, chromosome and DNA alterations, and the toxicity of available cryoprotectants continue to be responsible for significant rates of oocyte destruction (Johnson *et al.*, 1988; Vincent & Johnson, 1999; Woods *et al.*, 2004), cryopresevation of mature oocytes has been highlighted as a standardized technique by several reports in the last decade.

The importance of the technique is that it provides solution to most of the ethical, legal and religious questions involved in embryo freezing. Thus, it is particularly interesting for women without a partner or who do not accept fertilization with donor sperm, or those who are against embryo freezing for personal reasons. Above all, women who opt to preserve fertility may have ensured her write to decide not only when to have her child, but also with who, without a medical emergency dictating this decision. In addition, this technique plays an important role among the options for the preservation of fertility in adolescents, since them usually have no defined partner with whom they want to constitute a family.

Reported implantation and pregnancy rates vary from 10% to 60%, and 30% to 60%, respectively (Cobo *et al.*, 2013). Safety of transferred cryopreserved, thawed-warmed fertilized oocytes has been frequently reported. Although there is still a small number of children born after such a strategy, fetal wellbeing, birth defects and health outcomes seem to be as frequent as those reported in naturally conceived newborns (Noyes *et al.*, 2009). Long-term follow-ups of children are expected to fill in the gaps of knowledge in a brief future. Despite all the controversy in relation to this technique in 2012, the American Society for Reproductive Medicine (ASRM), recognized the efficiency of oocyte cryopreservation, and determined that it would be no longer an experimental technique, but also one more option within the reproduction procedures available (The Practice Committee of the American Society for Reproductive Medicine, 2013).

The limitations presented by this technique are the same for embryo cryopreservation, excepted for the need for a partner ou semen donor, with all its psychological and ethical implications.

### 5. Cryopreservation of Immature Oocytes

This method emerged as an alternative to the freezing of mature oocytes since immature oocytes are theoretically more resistant than mature oocytes to the freezing process by being more undifferentiated, by the absence of a spindle and by having chromosomes protected by a nuclear membrane. Some authors propose the puncture of still immature oocytes and the application of in vitro maturation (IVM) techniques associated with the cryopreservation of these gametes in order to minimize the time of induction and reduce the costs and side effects of controlled ovarian hyper stimulation (Cha *et al.*, 1998). However, results regarding oocyte survival and maturation up to metaphase II do not seem to be very encouraging. This method will not be valid until IVM of oocytes becomes a routine procedure (Kim, 2006).

### 6. Cryopreservation of Ovarian Tissue

Cryopreservation of ovarian tissue has been the subject of numerous studies, with recent data showing complete recovery of gonadal function after autograft of frozen-thawed ovarian tissue in rodents, sheep, primates (Oehninger, 2005) and humans (Donnez *et al.*, 2004), as well as twenty four human live births have been reported from this technique (Donnez *et al.*, 2004; Donnez *et al.*, 2012; Meirow *et al.*, 2005; Isachenki *et al.*, 2012; Revelli et al, 2013; Callejo *et al.*, 2013).

Once reproductive results are still considered scarse and limited, it is considered an experimental technique. In study protocols, cryopreservation of ovarian tissue serves to specific groups of patients for whom the other techniques are not recommended, such as prepubertal patients whose gonads are not yet under control of the hypothalamus-pituitary axis; patients with estrogen-dependent neoplasias with strict contra-indication to ovarian hyperstimulation; and women with malignancies that require an immediate approach, for whom the time needed for ovulation induction would have impact on the prognostic of the oncological disease.

Particularly from this perspective, the advantage of the collection of ovarian cortical tissue for cryopreservation is that it can be performed at any time during the menstrual cycle by videolaparoscopy, permitting the acquisition of hundreds of thousands primordial follicles (Poirot *et al.*, 2002), with the advantage that the antineoplasic therapy can be started two or three days latter, if no surgical complications occur.

The option of freezing ovarian cortex is based on early immature follicles resistance to cryotoxicity (Oktay *et al.*, 1997), which may result from peculiar characteristics of inactive follicles, such as reduced metabolism, absence of the zona pellucida and greater ease of cryoprotector penetration due to the smaller follicular size (Oktay *et al.*, 1998). Studies show that combining slow freezing and rapid thawing seem to be the most favorable protocol (Hovatta, 2004; Campos *et al.*, 2011). However up to date no consensus has been reached regarding which protocol would be the most appropriate to cryopreserve the ovarian tissue. In the slow freezing technique low concentrations of cryoprotectants are used in the attempt to prevent the formation of intracellular ice crystals (Maltaris *et al.*, 2006). But all the water may not to be totally eliminated from inside the ovarian sample, leading to ice crystals formation what can cause damage.

Recent studies in favor of ovarian tissue vitrification were published using nonhuman primates (Ting *et al.*, 2011; Ting *et al.*, 2012; Ting *et al.*, 2013). And the first human livebirth derived from ovarian tissue vitrification and ART was recently reported (Kawamura *et al.*, 2013). Moreover questions related to quantity of tissue to be cryopreserved, the use of components and procedures that might reduce tissue ischemia and the choice of cryopreservation protocol are still being strongly discussed.

A major concern of the scientific community regarding ovary freezing is the establishment of criteria for the evaluation of frozen tissue after thawing. It was demonstrated that although some loss in viability is observed after cryopreservation, the duration of cryopreservation does not interfere with the morphology or steroidogenic capacity of thawed ovarian tissue (Campos *et al.*, 2011).

The great limitation of the method still is the risk of metastasis’ reimplantation when grafting the thawed tissue, which should lead to recurrence of cancer. Studies in mouse model have been reported indicating a potential risk for lymphoma development in healthy mouse transplanted with ovarian fragments from mice with lymphoma (Shaw *et al.*, 1996). It is important to highlight that the type of tumor also influence in this risk, once some tumors are more likely to spread to the ovary than others (Sönmezer *et al.*, 2005).

In order to minimize all those risks, a big effort has been made to develop an efficient technique to mature extreme immature follicles (primordial, primary and secondary) isolated from cryopreserved ovarian tissue. The results are also limited, and although healthy new born mice have been reported after isolated secondary follicle culture (Xu *et al.*, 2006), no mature oocytes have been obtained from cultured human follicles.

## ETHICS

The possibility of conceiving a child after a cancer improves self-esteem and may contribute to a better acceptance of treatment and its adverse effects. There is no doubt that the greatest benefits are psychoemotional since the impossibility of biological maternity generates anguish and disorders of social interaction (Dudzinski, 2004). However, the risk of lack of success should be fully explained since currently available strategies do not guarantee that gametes and gonads will be protected by hormonal and surgical interventions or will resist cryopreservation techniques (The Ethics Committee of the American Society for Reproductive Medicine, 2013).

A multidisciplinary approach is essential, with an integrated action of physicians and psychologists in order to permit conscious decision making since the vulnerability of and enormous pressure suffered by everybody when a diagnosis of cancer is made directly interfere with their ability to understand and accept.

Patient safety is the major ethical concern when proposing to preserve ovarian function in cancer patients, since it would be futile to try to preserve fertility if the patient does not have the physical well-being necessary for a healthy maternity.

Regarding the preservation of embryos and oocytes, the risks related to ovarian stimulation should be considered. These risks range from the worsening of estrogen-dependent neoplasias to treatment postponement and to the controversial predisposition to the development of ovarian cancer. Ovarian hyperstimulation syndrome should also be kept in mind and proper care should be taken to prevent the systemic consequences of this process from interfering with the general pre-treatment condition of the patient. Within the experimental context, in which uncertainty prevails, it is extremely important to inform the patient or the person legally responsible for her and the relatives involved in the process about the limits of the success of the procedure, and also to document the issue of free and informed consent. The principles of autonomy, of benefiting the patient and not causing any harm should guide the chosen strategy, also respecting the opinion of a child if she is in a position to understand what is occurring.

Questions related to the period of storage of frozen tissues in banks, as well as their posthumous use and donation to recipients or for research continue to be ethical dilemmas. Facing the death of the biological mother, which are the rights of the biological father on preserved embryos? And in such a case, would it be ethical to transfer the embryos to a new partner of the biological father? How to contextualize the family of deceased biological mother? Despite efforts to regulate this new field of medicine, only its evolution will allow us to obtain replies.

## PERSPECTIVES

Growing ovarian follicles in vitro is a promising strategy for fertility preservation. Since primordial follicles are the most abundant in the ovary, are found in all females and are fairly resistant to low temperatures and antineoplastic drugs (Hornick *et al.*, 2012), the comprehension on how to use them with that objective would mean a very important advance in oncofertility, especially for children, adolescent and young female adult cancer patients.

Human secondary follicles have already been isolated from cancer patients gonads, becoming functional and developing from the early secondary stage to the antral stage in vitro, demonstrating healthy somatic cell-oocyte connections and production of hormones (Xu *et al.*, 2009a; Barrett *et al.*, 2010).

Success has been also achieved in non-human primates with in vitro secondary follicle culture collected from fresh and cryopreserved tissue.

They grew from secondary to antral stage and showed steroid hormone production (Xu *et al.*, 2009b; Xu *et al.*, 2010; Xu *et al.*, 2011; Ting *et al.*, 2011; Ting *et al.*, 2012; Ting *et al.*, 2013).

This experimental technique is also evaluating the culture of primordial and primary follicles in humans with results showing both follicles and oocytes growing the double in size (Amorim *et al.*, 2009; Bian *et al.*, 2013; Vanacker *et al.*, 2013).

Culture systems that support the growth of individual human preantral follicles still need to be refined in many ways. Many groups are working on the optimization of this culture system. The in vitro follicle maturation after cryopreservation of the ovarian tissue would be an excellent alternative to preserve the fertility of cancer patients, with low coast, low risk and a huge population of germ cells.
